# Interleukin 31 receptor alpha augments muscarinic acetylcholine receptor 3-driven calcium signaling and airway hyperresponsiveness in asthma

**DOI:** 10.21203/rs.3.rs-2564484/v1

**Published:** 2023-02-15

**Authors:** Santoshi Akkenepally, Dan JK Yombo, Sanjana Yerubandi, Bhanuprakash R. Geereddy, Francis X. McCormack, Satish K Madala

**Affiliations:** 1 Division of Pulmonary, Critical Care and Sleep Medicine, Department of Internal Medicine, University of Cincinnati, Cincinnati, Ohio USA.; 2 Division of Pulmonary Medicine, Department of Pediatrics, Cincinnati Children’s Hospital Medical Center, Cincinnati, Ohio USA.; 3 Division of Biochemistry, National Institute of Nutrition, Hyderabad, Telangana, India.

**Keywords:** allergic asthma, airway hyperresponsiveness, interleukin 31, calcium signaling, lung

## Abstract

Asthma is a chronic inflammatory airway disease characterized by airway hyperresponsiveness (AHR), inflammation, and goblet cell hyperplasia. Both Th1 and Th2 cytokines, including IFN-γ, IL-4, and IL-13 have been shown to induce asthma; however, the underlying mechanisms remain unclear. We observed a significant increase in the expression of IL-31RA, but not its cognate ligand IL-31 during allergic asthma. In support of this, IFN-γ and Th2 cytokines, IL-4 and IL-13, upregulated IL-31RA but not IL-31 in airway smooth muscle cells (ASMC). Importantly, the loss of IL-31RA attenuated AHR but had no effects on inflammation and goblet cell hyperplasia in allergic asthma or mice treated with IL-13 or IFN-γ. Mechanistically, we demonstrate that IL-31RA functions as a positive regulator of muscarinic acetylcholine receptor 3 expression and calcium signaling in ASMC. Together, these results identified a novel role for IL-31RA in AHR distinct from airway inflammation and goblet cell hyperplasia in asthma.

## INTRODUCTION

Asthma is a common chronic inflammatory disease that affects more than 300 million people worldwide. Asthma is associated with significant health and economic burdens and impairs both quality of life and productivity^1,2^. Despite advances in understanding the disease physiopathology and the development of anti-inflammatory therapeutic interventions, the incidence of asthma, including uncontrolled and severe forms, continues to increase. The disease is characterized by episodes of airflow obstruction that are associated with high mortality when severe and unreversed^3^. The primary clinical manifestations in the pathophysiology of chronic asthma include airway hyperresponsiveness (AHR), inflammation, goblet cell hyperplasia with mucus production, and fibrotic airway remodeling^4^. AHR is one of the cardinal features of asthma, but AHR can also be present in other airway disorders such as cystic fibrosis and COPD^5,6^. Several published studies have implicated excessive airway smooth muscle contraction, airway inflammation, dysregulated neural control, and fibrotic airway remodeling as key mechanisms underlying AHR. Therefore, identifying a common pathway through which a number of these potential mechanisms operate could facilitate the development of novel therapies for asthma and other chronic obstructive lung diseases. Traditionally, asthma was classified based on definable phenotypes, often based on features such as a clearly identifiable allergic component; however, more recently,^7^ “endotypes” delineated by cellular and molecular pathophysiologic mechanisms that reflect mechanistic heterogeneity have been established. Currently, they include immune response endotypes driven not only by Th2 but also by Th1 (IFNγ) or Th17 (IL-17) in severe forms of asthma which are associated with eosinophilia and neutrophilia^8–13^. Th2 cytokines, IL-4 and IL-13, which are elevated in the lungs and serum of asthmatic patients, are now well-established drivers of AHR and airway inflammation in asthma^14–16^. IL-13 is a major cytokine responsible for goblet cell hyperplasia, tissue remodeling, and bronchospasm. Even in the absence of an allergen exposure or inflammation, IL-13 alone is sufficient to induce AHR and airway inflammation in mice^17,18^. IL-4 is crucial in the regulation of Th2 cell proliferation as well as the antibody class switch to produce IgE, and recent studies highlight a shared role of this cytokine with IL-13 in the induction of AHR and inflammation through their common receptor type II IL-4Rα, as well as through type I IL4Rα^19,20^. Clinical trials that neutralize the common IL-4Rα receptor for these Th2 cytokines have shown improvements in AHR and inflammation in both allergic asthma and atopic dermatitis patients^21,22^. However, these therapies and other more conventional treatments for asthma have not been effective against low type 2 or non-type 2 endotypes and severe asthma with a mixed Th1/Th2 cytokine phenotype^23^. A better understanding of the mechanisms that regulate AHR and therapeutic resistance in these specific conditions may of help to develop alternative therapeutic approaches to control more atypical asthma endotypes.

IL-31 is a Th2-related cytokine implicated in tissue remodeling in chronic lung and skin diseases^24,25^, including allergic asthma, atopic dermatitis (AD), fibrosis, and itch-associated conditions^24–28^. IL-31 specifically binds to a heterodimeric receptor complex formed by the IL-31 receptor alpha (IL-31RA) and oncostatin-M specific receptor beta (OSMRβ) and signals through MAPK, JAK, STAT1, STAT3, and STAT5^29,30^. IL-31 is predominantly produced by hematopoietic cells, whereas its receptor, IL-31RA, is predominantly expressed in non-hematopoietic epithelial and smooth muscle cells^31–34^. Although studies have reported an association between the expression of both IL-31 and IL-31RA with severe asthma^35,36^, the focus of most prior studies has been on tissue remodeling observed in diseases such as pulmonary fibrosis. For example, we recently published the absence of IL-31RA signaling had a protective effect against the impairment of lung function in the bleomycin mouse model of pulmonary fibrosis^37^. Also, studies from our lab and others have shown that Th2 cytokines, IL-4 and IL-13, augment the expression of IL-31RA in immune cells, including macrophages^21,38^. However, the role of the IL-31-IL-31RA axis in the induction of AHR or airway inflammation during allergic asthma remains unexplored.

In this study, we evaluated the effects of the IL-31-IL-31RA axis on allergen-induced AHR, inflammation, and goblet cell hyperplasia in mice with wild-type and IL-31RA deficient gene expression. We demonstrated a significant increase in the expression of IL-31RA, but not IL-31, and both Th1 and Th2 cytokines (IFN-γ, IL-4, and IL-13) in two alternative mouse models of allergic asthma. The loss of IL-31RA was sufficient to attenuate both allergen and cytokine (IFN-γ, and IL-13)-induced AHR but had limited or no effect on inflammation and goblet cell hyperplasia. We identified a novel mechanism underlying IL-31RA-driven AHR involving heightened muscarinic acetylcholine receptor 3 (CHRM3) expression and calcium signaling that together induces ASMC contraction. The finding that the IL-31RA-CHRM3 axis augments calcium signaling and ASMC contraction may facilitate the design of therapies with greater efficacy to target AHR downstream of both Th1/Th2 cytokines in asthma.

## RESULTS

### Upregulation of IL-31RA promotes airway hyperresponsiveness

**Figure 1.**
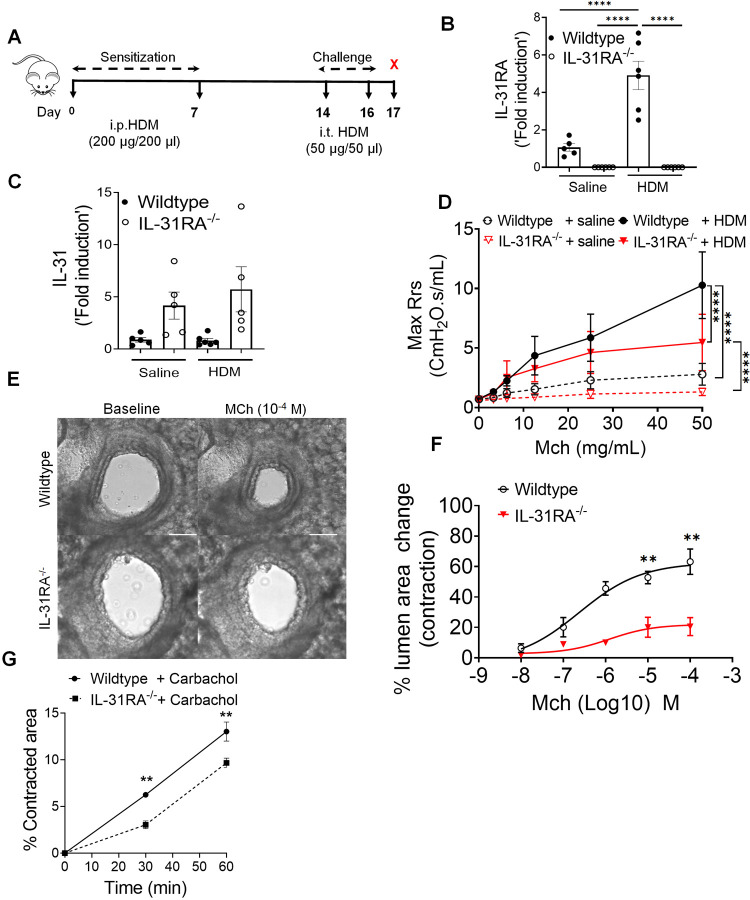
Loss of IL-31RA attenuates house dust mite (HDM)-induced airway hyperresponsiveness. **(A)** Schemata of HDM-induced allergic asthma model. **(B and C)** Quantification of IL-31RA and IL-31 transcripts in the lungs of wild-type and IL-31RA^−/−^ mice treated with HDM or saline. Data shown as mean ± SEM, n = 5–6/group, one-way ANOVA test, * p < 0.05. **(D)** Measurement of resistance with increasing doses of methacholine (MCh) in wild-type and IL-31RA^−/−^ mice treated with saline or HDM using FlexiVent. Data are shown as mean ± SEM, n = 7–8/group. A two-way ANOVA test, * p < 0.05. **(E)** Representative images of precision cut lung sections (PCLS) from the lungs of wild-type and IL-31RA^−/−^ mice treated with MCh (10^−4^ M) or prior to the treatment (baseline), scale bar 150 μm. **(F)** The percent of contraction of airways with increasing doses of MCh compared to the baseline area of airways between wild-type and IL-31RA^−/−^ mice. A two-way ANOVA test; n = 3–8 per group. * p < 0.05. **(G)** The percent contraction of collagen gels embedded with airway smooth muscle cells from wild-type and IL-31RA^−/−^ mice in culture media and treated with carbachol (10 μM) for 60 minutes. N = 3/group. Data shown as means mean ± SEM, two-tailed Student’s t-test, *p < 0.05

Loss of type II IL-4 receptor signaling attenuates the pathological features of asthma and expression of IL-31RA during *Schistosoma mansoni* soluble egg antigen (SEA)-induced allergic asthma^11,20,38–40^. We hypothesized that in a house dust mite (HDM)-induced model of allergic asthma, the loss of IL-31RA would block the development of some, if not all, pathological features of allergic asthma. To elucidate the role of IL-31RA in allergic asthma, IL-31RA knockout and wild-type mice were sensitized twice with intraperitoneal (i.p.) HDM followed by intratracheal (i.t.) challenge with HDM a week later for two consecutive days (Fig 1A). The control mice were sensitized and challenged with saline. As expected, the expression of IL-31RA was not detected in IL-31RA knockout (IL-31RA^−/−^) mice (Fig 1B). Sensitization and challenge with HDM or saline did not modify the gene expression of IL-31 in either wild-type or IL-31RA^−/−^ mice (Fig 1C), but significantly elevated IL-31RA in wild-type mice compared to saline-treated mice (Fig 1B). The loss of IL-31RA was sufficient to attenuate dose-dependent methacholine (MCh)-induced AHR in IL-31RA^−/−^ mice compared to wild-type mice sensitized and challenged with HDM (Fig 1D). To assess whether the observed decrease in AHR was due to impairment of airway contraction, we prepared precision-cut lung slices (PCLS) from wild-type and IL-31RA knockout mice and tested dose-dependent MCh-induced airway contraction in real time. MCh-induced airway contraction was decreased in PCLS prepared from the lungs of IL-31RA^−/−^ mice compared to that in wild-type mice (Fig 1E and 1F). Furthermore, we used a collagen gel contraction assay to measure carbachol-induced contraction of airway smooth muscle cells (ASMC) isolated from the trachea of wild-type and IL-31RA^−/−^ mice. Collagen gels embedded with wild-type ASMC showed higher contraction compared to IL-31RA^−/−^ ASMC within 30 minutes in floating collagen gels (Fig 1G).

### Airway inflammation and goblet cell hyperplasia are IL-31RA independent

**Figure 2.**
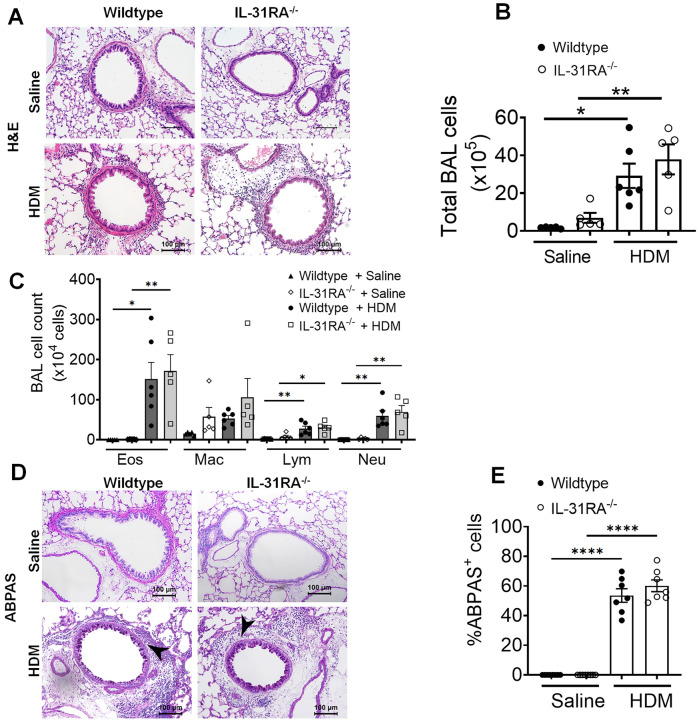
Loss of IL31RA has no effect on house dust mite (HDM)-induced airway inflammation and goblet cell hyperplasia. **(A)** Representative images of hematoxylin and eosin -stained lung sections from wild-type and IL-31RA^−/−^ mice treated with saline or HDM. Images were taken at 20x magnification, scale bar 100 μm. **(B and C)** Total bronchoalveolar lavage (BAL) cell number and the differential cell count of BAL cells of wild-type and IL-31RA^−/−^ mice treated with saline or HDM. Eos, eosinophils; Mac, macrophages; Lym, lymphocytes; and Neu, neutrophils. **(D)** Representative images of alcian blue periodic acid shiff(ABPAS) staining of lung sections from wild-type and IL-31RA^−/−^ mice treated with saline or HDM. Images were taken at 20x magnification, scale bar 100 μm. **(E)** The percent of ABPAS-positive cells normalized to total cell in the airways of wild-type and IL-31RA^−/−^ mice treated with saline or HDM. Data are shown as means ± SEM. One-way ANOVA was used, with n = 7–8/group. * p < 0.05.

Mice primed and challenged with HDM develop strong Th2 responses, peribronchial inflammation, and goblet cell hyperplasia^41,42^. To determine whether IL-31RA was critical for the development of airway inflammation, we stained the lung sections of wild-type and IL-31RA^−/−^ mice challenged with HDM or saline with hematoxylin and eosin (H&E) (Fig 2A). As expected, the HDM challenge induced robust airway inflammation with immune cell infiltration in wild-type mice, as confirmed by quantitative measurement of the total bronchoalveolar lavage (BAL) cells (Fig 2B & 2C). Notably, the IL-31RA^−/−^ mice that were challenged with HDM developed airway inflammation similar to wild-type mice, indicating no effect of IL-31RA deficiency on allergic inflammatory response in airways. We further investigated whether the loss of IL-31RA had any effect on the infiltration of specific immune cell types into the airspaces by differential counting of the immune cell types in BAL. Consistent with the above findings, we observed no significant differences in the number of eosinophils, macrophages, lymphocytes, and neutrophils between wild-type and IL-31RA^−/−^ mice challenged with either saline or HDM (Fig 2C).

Accumulation of mucus-secreting goblet cells or goblet cell hyperplasia is another important hallmark of allergic asthma observed in both human and animal models of allergic asthma^43–45^. To determine whether there were differences in goblet cell hyperplasia, we performed alcian blue periodic acid-Schiff (ABPAS) staining to detect mucus-producing goblet cells in lung sections. Both wild-type and IL-31RA^−/−^ mice displayed increased goblet cell hyperplasia when challenged with HDM compared to unchallenged naïve animals (Fig 2D and 2E). These findings suggest that IL-31RA deficiency contributes to the development of AHR but has no significant effect on airway inflammation and mucus production in HDM-induced allergic asthma.

**Figure 3.**
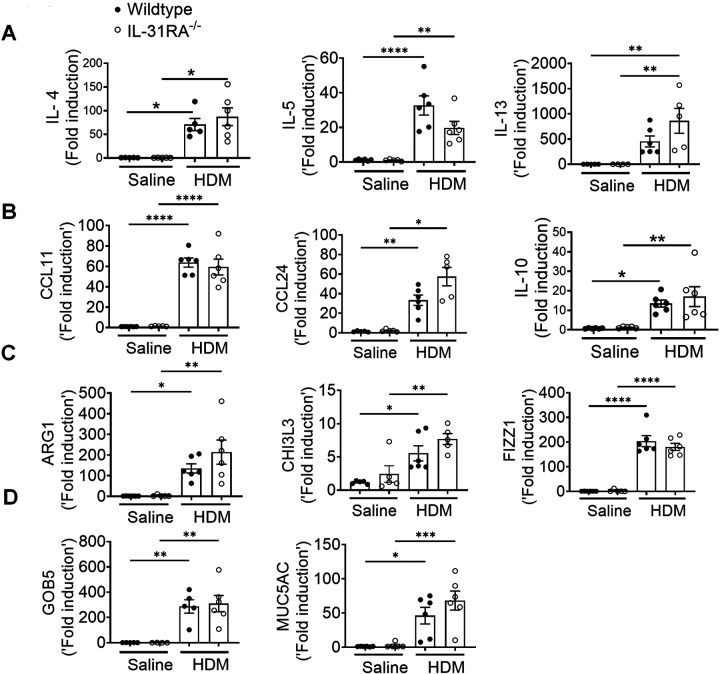
Loss of IL-31RA has no effect on Th2 responses and goblet cell hyperplasia during house dust mite (HDM)-induced allergic asthma. **(A)** Quantification of Th2 cytokine transcripts including IL-4, IL5, and IL-13 in the whole lung tissue of wild-type and IL-31RA^−/−^ mice treated with saline or HDM using RT-PCR. **(B)** Quantification of chemokines and inflammatory cytokines including CCL11, CCL24, and IL-10 in the whole lungs of wild-type and IL-31RA^−/−^ mice treated with saline or HDM using RT-PCR. **(C)** Quantification of Th2 response-associated gene transcripts including CHI3L3, ARG1, and FIZZ1 in the total lungs of wild-type and IL-31RA^−/−^ mice treated with saline or HDM using RT-PCR. **(D)** Quantification of GOB5 and MUC5AC gene transcripts in the whole lungs of wild-type and IL-31RA^−/−^ mice treated with saline or HDM using RT-PCR. Data are shown as mean ± SEM and representative of two independent experiments with n=5–6/group. One-way ANOVA was used. * p < 0.05.

IL-31RA functions as a negative regulator of Th2 responses in the lungs^46^ However, the role of IL-31RA in the development of Th2 responses during allergic asthma remains unclear. To determine whether there are alterations in the expression of asthma-associated genes in the absence of IL-31RA, we measured the transcript levels of Th2 cytokines, inflammatory cytokines, Th2 responses, and goblet cell hyperplasia-associated genes. As expected, we observed a significant increase in Th2 cytokine gene expression, including IL-4, IL-5, and IL-13, in wild-type mice challenged with HDM compared to that with saline (Fig 3A). However, HDM-induced increase in Th2 cytokine expression was similar in the lungs of wild-type and IL-31RA^−/−^ mice, suggesting that the absence of IL-31RA had no effect on HDM-induced Th2 cytokine expression. Similarly, the loss of IL-31RA had no effect on the expression of chemokine and cytokine genes (CCL11, CCL24, and IL-10) and Th2 response-associated genes, including ARG1, CHI3L3, and FIZZ1 (Fig 3B & 3C). Consistent with ABPAS staining, we observed no effect of IL-31RA deficiency on HDM-induced expression of GOB5 and MUC5AC (Fig 3D). IL-6 and oncostatin M (OSM), together with IL-31, are members of the IL-6 family of cytokines known to regulate AHR in allergic asthma^47–49^. Thus, we explored the levels of OSM and IL-6 in the lungs of wild-type and IL-31RA^−/−^ mice (Fig S1). We observed no significant differences in the expression of IL-6 and OSM between wild-type and IL-31RA^−/−^ mice treated with either saline or HDM, suggesting that the absence of IL-31RA did not affect the expression of OSM and IL-6.

### IL-31RA is essential to induce AHR during SEA-induced allergic asthma

**Figure 4.**
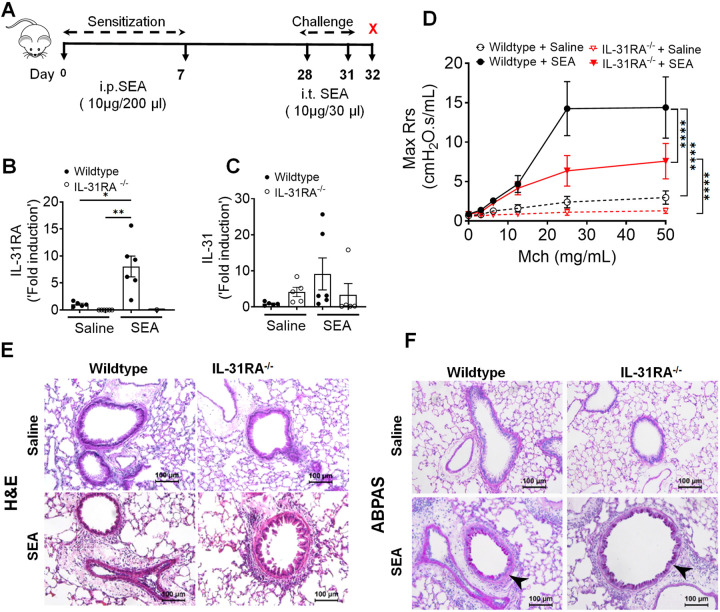
Loss of IL31RA attenuates AHR but not inflammation and goblet cell hyperplasia during SEA-induced allergic asthma. **(A)** Schemata of SEA-induced allergic asthma model. **(B and C)** Quantification of IL-31RA and IL-31 transcripts in the lungs of wild-type and IL-31RA^−/−^ mice treated with house dust mite (HDM) or saline. Data shown as mean ± SEM, n = 5–6/group, one-way ANOVA test, *p < 0.05. **(D)** Measurement of resistance with increasing doses of methacholine (MCh) in wild-type and IL-31RA^−/−^ mice treated with saline or HDM using FlexiVent. Data are shown as mean ± SEM, n = 5–6/group. The above data is representative of two independent experiments with similar results. A two-way ANOVA test for multiple comparisons was used. * p<0.01. **(E)** Representative images of hematoxylin and eosin-stained lung sections from wild-type and IL-31RA^−/−^ mice treated with saline or SEA. Images were taken at 20x magnification, scale bar 100 μm. **(F)** Representative images of alcian blue periodic acid shiff staining of lung sections from wild-type and IL-31RA^−/−^ mice treated with saline or SEA. Images were taken at 20x magnification, scale bar 100 μm.

To further establish the role of IL-31RA in allergic asthma, we used an alternative mouse model of SEA-induced allergic asthma. Similar to HDM, extract of *Schistosoma mansoni* is a potent inducer of AHR, airway inflammation, and Th2 immune responses even without the use of an adjuvant^28,50,51^. As shown in the schematic, wild-type and IL-31RA^−/−^ mice were sensitized twice and consecutively challenged with SEA to induce allergic asthma (Fig 4A). The absence of IL-31RA gene expression was confirmed by measuring transcripts in the lungs of mice challenged with SEA or saline, and no IL-31RA expression was detected in IL31RA^−/−^ mice (Fig 4B). Consistent with the findings using the HDM model, sensitization and challenge with SEA resulted in a significant increase in the gene expression of IL-31RA but not IL-31 in the wild-type mice (Fig 4B & 4C).

To assess whether the loss of IL-31RA attenuates SEA-induced AHR, we measured MCh-induced AHR in both wild-type and IL-31RA^−/−^ mice sensitized and challenged with either saline or SEA (Fig 4D). Loss of IL-31RA was sufficient to attenuate MCh-induced AHR in SEA-challenged mice (Fig 4D). To evaluate the changes in airway inflammation, lung sections of wild-type and IL31RA^−/−^ mice challenged with SEA or saline were stained with H&E. As observed in the HDM model, SEA challenge induced robust airway inflammation with immune cell infiltration in both wild-type and IL-31RA^−/−^ mice (Fig 4E). Similarly, the airways of IL-31RA^−/−^ mice that were challenged with SEA showed an accumulation of mucus-producing goblet cells similar to wild-type mice, as seen in ABPAS-stained lung sections (Fig 4F).

**Figure 5.**
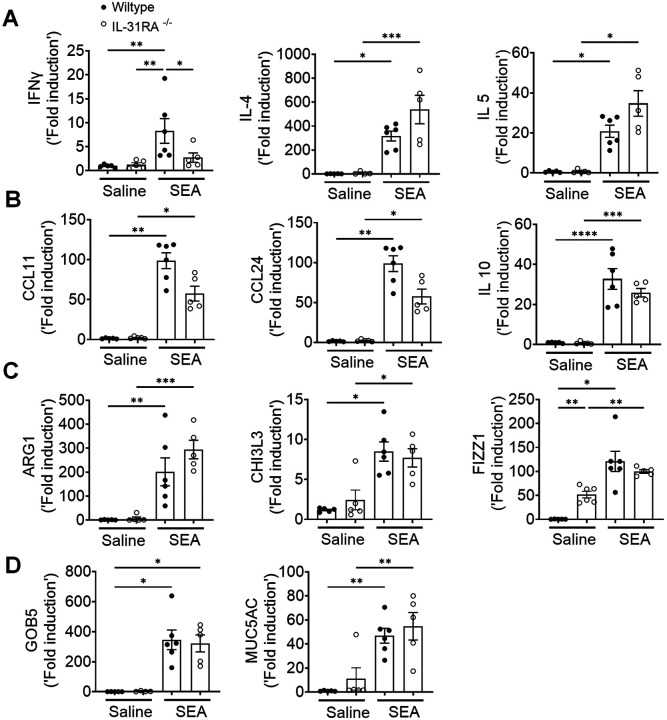
Loss of IL31RA has no effect on Th2 responses and goblet cell hyperplasia during SEA-induced allergic asthma. **(A)** Quantification of IFN-γ, IL-4, IL-5, and IL-13 in the whole lungs of wild-type and IL-31RA^−/−^ mice treated with saline or SEA using RT-PCR. **(B)** Quantification of CCL11, CCL24, and IL-10 in the whole lungs of wild-type and IL-31RA^−/−^ mice treated with saline or SEA using RT-PCR. **(C)** Quantification of Th2 response-associated genes including CHI3L3, ARG1, and FIZZ1 in the whole lungs of wild-type and IL-31RA^−/−^ mice treated with saline or SEA using RT-PCR. **(D)** Quantification of GOB5 and MUC5AC gene transcripts in the whole lungs of wild-type and IL-31RA^−/−^ mice treated with saline or SEA using RT-PCR. Data are shown as means ± SEM and representative of two independent experiments with n=5–6/group. One-way ANOVA was used. * p < 0.05.

To determine whether IL-31RA-induced AHR is regulated by asthma-associated gene networks, we measured the expression of Th1 and Th2 cytokines. The expression of IFN-γ, IL-4, and IL-5 was elevated in both wild-type and IL-31RA^−/−^ mice following sensitization and challenge with SEA (Fig 5A). Similarly, the transcripts of genes associated with inflammation (CCL11, CCL24, and IL-10) and Th2 responses (ARG1, CHI3L3, and FIZZ1) remained elevated in IL-31RA^−/−^ mice, and no differences were observed when compared to wild-type mice treated with SEA (Fig 5B & 5C). The gene transcripts associated with goblet cell hyperplasia were elevated in both wild-type and IL-31RA^−/−^ mice treated with SEA (Fig 5D). In summary, in the two alternative mouse models of allergic asthma, the absence of IL-31RA altered AHR but did not appear to affect airway inflammation, Th2 responses, or goblet cell hyperplasia.

### IL-31 is dispensable to induce AHR, inflammation, and Th2 responses

**Figure 6.**
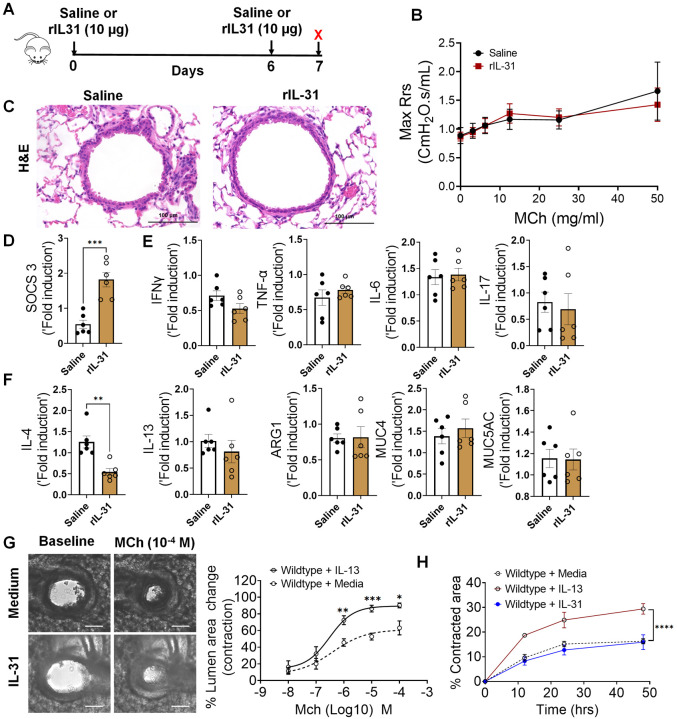
IL-31 is dispensable for the induction of AHR, inflammation, and Th2 responses. **(A)** Schemata showing intratracheal administration of IL-31 or saline in wild-type mice. **(B)** Measurement of resistance with increasing doses of methacholine (MCh) in wild-type mice treated with saline or IL-31 using FlexiVent. Data are shown as mean ± SEM, n = 6/group. The data is representative of two independent experiments with no statistical significance between groups. **(C)** Representative images of hematoxylin and eosin -stained lung sections from wild-type mice treated with IL-31 or saline. Images were captured at 20x magnification, scale bar 100 μm. **(D)** Quantification of IL-31-induced SOCS3 gene expression in the whole lung tissue of wild-type mice treated with saline or IL-31. Unpaired two-tailed Student’s t-test was used. ***P < 0.001. **(E and F)** Quantification of inflammation-associated gene transcripts *IFN-γ, TNFα, IL-6,* and *IL-17* and Th2-associated gene transcripts including *IL-4, IL-13, ARG1, MUC4,* and *MUC5AC* in whole lungs of wild-type mice treated with saline or IL-31. Unpaired two-tailed Student’s t-test was used and no significance found between groups, n=6/group. **(G)** Representative images of precision cut lung sections (PCLS) from wild-type mice treated with or without IL-31 (500 ng/ml) for 24 h. Airway contractility was measured in response to MCh (10^−4^ M) compared to baseline diameter. The percent of airway lumen area contraction with increasing doses of MCh was calculated for saline and IL-31-treated PCLS from wild-type mice. Two-way ANOVA test, n=5–8/group. **(H)** The percent contraction of collagen gels embedded with airway smooth muscle cells from wild-type mice that were treated with media, IL-13 (50 ng/ml) or IL-31 (500 ng/ml). The percent contraction was measured at different time points compared to baseline. Two-way ANOVA was used, n=4/group.

IL-31RA is a cognate binding receptor of IL-31 that recruits OSMRβ to form a high-affinity binding receptor complex that signals through the JAK/STAT pathway^29,30^. Previous studies have demonstrated that increased IL-31 signaling through IL-31RA can result in uncontrolled inflammation and tissue remodeling in multiple tissues, including the lungs and skin^25,26^. However, we observed no significant increase in IL-31 levels in the lungs during HDM- and SEA-induced allergic asthma. To assess the potential pathological effects of IL-31 in asthma, the lungs of wild-type mice were intratracheally treated with saline or IL-31, and changes in AHR and inflammation were assessed (Fig 6A). Notably, we observed no significant changes in AHR between saline and IL-31 treated wild-type mice (Fig 6B). Similarly, we observed no significant changes in tissue inflammation as assessed by H&E-stained lung sections of saline and IL-31 treated wild-type mice (Fig 6C). To confirm the signaling effects of IL-31, we measured the expression of the IL-31-driven gene suppressor of cytokine signaling 3 (*SOCS3)* in the lungs of mice treated with saline and IL-31. Previously published studies have shown that IL-31 upregulates *SOCS3*^52^; similarly, the expression of *SOCS3* was significantly upregulated in lungs of IL-31-treated mice compared to the saline-treated mice (Fig 6D). To determine the effects of IL-31 on the expression of asthma-associated genes, we quantified the expression of genes associated with inflammation (*IFN-γ, TNF-α, IL-6, and IL-17*) and Th2 responses (*IL-4, IL-13, ARG1, MUC4, and MUC5AC*). Notably, we observed no significant changes in the expression of genes associated with either inflammation or Th2 responses (Fig 6E and 6F).

Furthermore, we evaluated airway contractility induced by IL-31 using PCLS. As demonstrated in previous studies, we observed a significant increase in the contractility of airways of wild-type mice treated with IL-13 compared to saline-treated mice in a dose-dependent manner with MCh (Fig S2). Conversely, we observed no significant effect of IL-31 on the contractility of wild-type airways exposed to MCh in comparison to media (Fig 6G). Furthermore, we observed no significant changes in the kinetics of contraction of collagen gels embedded with ASMC, which were treated with either media alone or IL-31. However, we observed a significant increase in the contraction of collagen gels embedded with ASMC and treated with IL-13 compared to that with media alone (Fig 6H). Thus, in contrast to IL-31RA, the results suggest that IL-31 is not involved in modifying AHR, inflammation, and Th2 responses in the lungs; the reduced AHR observed in the absence of IL-31RA could be due to other alternative mechanisms that need to be identified.

### Th2 cytokines upregulate IL-31RA to induce AHR with no effect on inflammation

**Figure 7.**
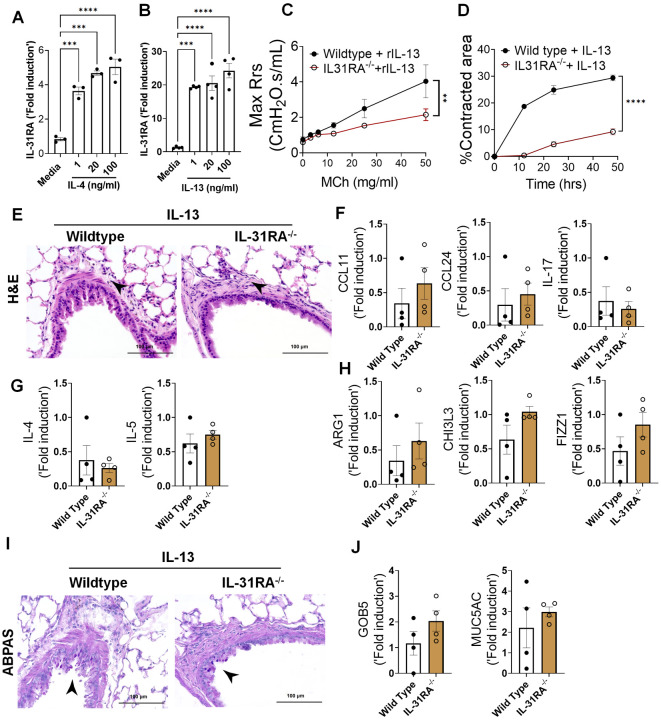
Th2 cytokines upregulate IL-31RA to induce AHR with no effect on inflammation and goblet cell hyperplasia. **(A and B)** Quantification of IL-31RA transcripts in mouse airway smooth muscle cells (ASMC) treated with increasing doses of IL-4 and IL-13 for 16 h. One-way ANOVA test was used, n = 3/group; * p<0.05. **(C)** Wild-type and IL-31RA^−/−^ mice were treated intratracheally with IL-13 on days D0 and D6 and resistance in the lungs was measured with increasing doses of methacholine (MCh) using FexiVent. Data are shown as mean ± SEM, n=4/group. Two-way ANOVA test was used, *p<0.05. **(D)** ASMC isolated from wild-type and IL-31RA^−/−^ mice were seeded into collagen gels and treated with IL-13 to measure the contraction of collagen gels after 48 hours. Two-tailed Student’s t-test was used, n=3/group; * p<0.05. **(E)** Representative images of hematoxylin and eosin -stained lung sections from wild-type and IL-31RA^−/−^ mice treated with IL-13. Images were captured at 20x magnification, scale bar 100 μm. **(F, G and H)** Quantification of inflammatory chemokines (*CCL11, CCL24 and IL-17*), Th2 cytokines (*IL-4* and *IL-5*), and Th2 response-associated genes (*ARG1, CHl3L3,* and *FIZZ1*) in the whole lungs of wild-type and IL-31RA^−/−^ mice treated with IL-13. Data are shown as mean ± SEM, n=4/group, Two-tailed Student’s t-test was used and no statistical significance observed between groups. **(I)** Representative images of alcian blue periodic acid shiff stained lung sections from wild-type and IL-31RA^−/−^ mice treated with IL-13. Images were captured at 20x magnification, scale bar 100 μm. **(J)** Quantification of goblet cell hyperplasia associated genes including *GOB5* and *MUC5AC* transcript levels in the total lungs of wild-type and IL-31RA^−/−^ mice treated with IL-13. Data are shown as means ± SEM, n=4/group, Two-tailed Student’s t-test was used and no statistical significance observed between groups.

Previous studies from our lab and others have shown that both IL-4 and IL-13 induce IL-31RA expression in macrophages and lung tissues via the type II IL-4 receptor and STAT6 signaling^38^. To determine whether IL-4 or IL-13 can induce IL-31RA expression in ASMC, we treated ASMC with increasing doses of IL-4 or IL-13 and quantified the expression of IL31RA. Consistent with our earlier findings in macrophages, the expression of IL-31RA significantly increased in ASMC treated with IL-4 or IL-13 compared to that with media (Fig 7A & 7 B). IL-13 is a potent inducer of AHR, inflammation, and goblet cell hyperplasia in mice^18^. To determine whether IL-13-induced AHR is IL-31RA dependent, we treated both wild-type and IL-31RA^−/−^ mice with IL-13 and assessed AHR and other pathological and molecular changes that are relevant to asthma. Notably, the loss of IL-31RA was sufficient to attenuate IL-13-induced AHR compared to wild-type mice exposed to increasing doses of MCh (Fig 7C). Similarly, the contraction of collagen gels embedded with ASMC from IL-31RA^−/−^ mice significantly reduced compared to wild-type mice with IL-13 treatment (Fig 7D). In contrast, H&E staining of lung sections suggested that the loss of IL-31RA had no effect on IL-13-driven airway inflammation in wild-type and IL31RA^−/−^ mice (Fig 7E). Quantitative assessment of inflammatory chemokines and cytokines suggested that the loss of IL-31RA had no effect on the expression of CCL11, CCL24, and IL-17 (Fig 7F). Similarly, we observed no defects in the expression of Th2 cytokines (IL-4 and IL-5) or genes associated with Th2 responses (ARG1, CHI3L3, and FIZZ1) in IL-31RA^−/−^ mice compared to wild-type mice treated with IL-13 (Fig 7G and 7H).

To evaluate whether IL-13-induced goblet cell hyperplasia was altered in the absence of IL-31RA, we assessed the accumulation of goblet cells in the airways and quantified the transcripts associated with goblet cell hyperplasia. As shown in figure 7I, goblet cell accumulation was similar between wild-type and IL31RA^−/−^ mice treated with IL-13. In support of this, the expression of *GOB5* and *MUC5AC* remained similar between IL-13 treated wild-type and IL31RA^−/−^ mice (Fig 7J). Thus, IL-31RA expression is essential to mediate IL-13-induced AHR but dispensable for inflammation and goblet cell hyperplasia.

### IFN-γ upregulates IL-31RA to induce AHR with no effects on inflammation

**Figure 8.**
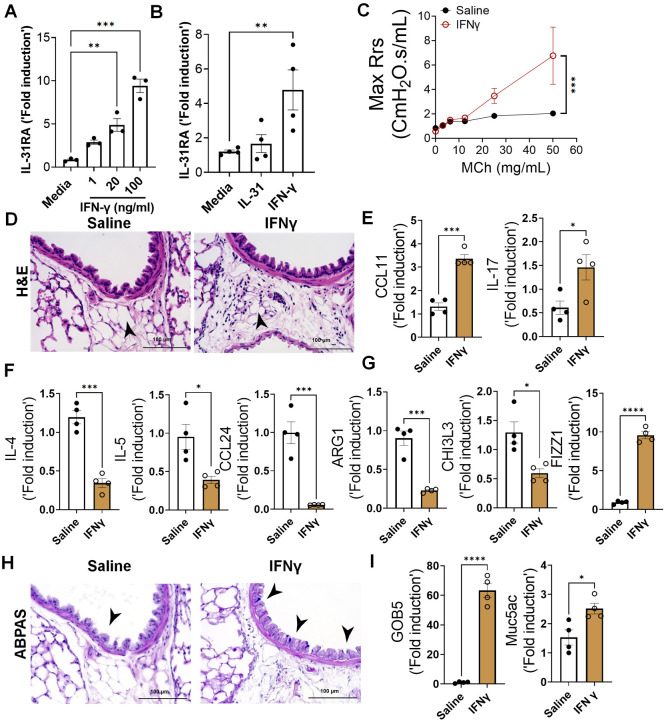
IFN-γ is a positive regulator of IL31RA expression, along with AHR, inflammation, and goblet cell hyperplasia. **(A)** Quantification of IL-31RA transcript levels in mouse airway smooth muscle cells (ASMC) treated with increasing doses of IFN-γ for 16 h. Data are shown as the mean ± SEM (n = 3/group). One-way ANOVA was used; *P < 0.05. **(B)** Quantification of IL-31RA transcript levels in human ASMCs treated with IL-31 (500 ng/ml) or IFN-γ (50 ng/ml) for 16 h. Data are shown as the mean ± SEM, n = 3–4/group. One-way ANOVA was used; *P < 0.05. (C) Wild-type mice were intratracheally treated with IFN-γ (5 μg) on days 0 and 6, and airway resistance with increasing doses of methacholine was measured on day 7. Data are shown as mean ± SEM, n = 4/group; two-way ANOVA was used, * P < 0.05. **(D)** Representative images of hematoxylin and eosin -stained lung sections from wild-type mice treated with saline or IFN-γ. Images were captured at 20x magnification, with a scale bar of 100 μm. **(E, F, and G)** Quantification of inflammatory chemokines (*CCL11* and *IL-17*), Th2 cytokines (*IL-4, IL-5, and CCL24)*, and Th2 response-associated genes, including *ARG1, CHl3L3*, and *FIZZ1*, in the lungs of wild-type mice treated with saline or IFN-γ. Data are shown as the mean ± SEM, n = 6/group. Two-tailed Student’s t-test was used; * P < 0.05. **(H)** Representative images of alcian blue periodic acid shiff -stained lung sections from wild-type mice treated with saline or IFN-γ. Images were captured at 20x magnification, scale bar = 100 μm. **(I)** Quantification of mucus-associated gene transcripts, including *GOB5* and *MUC5AC*, in the lungs of wild-type mice treated with saline or IFN-γ. Data are shown as the mean ± SEM (n = 6/group). Two-tailed Student’s t-test was used; * P < 0.05.

IFN-γ is a key Th1 cytokine implicated in elevated AHR in patients with severe asthma and those with mixed Th1/Th2 cytokine phenotypes^12,53^. To determine whether IFN-γ induces the expression of IL-31RA in ASMC, we isolated ASMC from the tracheas of wild-type mice and treated them with increasing doses of IFN-γ. The expression of IL-31RA is significantly increased by IFN-γ in a dose dependent manner (Fig 8A). A recent study suggested that IL-31 can also upregulate the expression of IL-31RA in human sensory neurons^54^. However, we observed no significant effect of IL-31 on the expression of IL-31RA in ASMC (Fig 8B). To determine the effects of IFN-γ on asthma phenotypes, we treated wild-type mice with saline or IFN-γ and measured the changes in AHR, inflammation, and goblet cell hyperplasia. We observed a significant increase in AHR in wild-type mice treated with IFN-γ compared to that with saline (Fig 8C). In addition, we observed a significant increase in peri-bronchial inflammation in wild-type mice treated with IFN-γ compared to that with saline (Fig 8D). To establish the quantitative gene expression changes induced by IFN-γ, we measured IRF1, IRF7, and STAT1 and observed a significant increase in the expression of IFN-γ-specific genes in the lungs (Fig S3).

To determine the effects of IFN-γ on asthma phenotypes, we measured the expression of genes associated with inflammation and Th2 responses. In support of the substantial inflammation observed after IFN-γ treatment, we observed a significant increase in CCL11 and IL-17 expression (Fig 8E). As anticipated, IFN-γ treatment resulted in negative regulation of Th2 cytokines and Th2 response-associated gene expression, including IL-4, IL-5, CCL24, ARG1, and CHI3L3, but had no effect on the expression of FIZZ1, which was significantly increased compared to the saline group (Fig 8F and G). Similar to IL-13, IFN-γ was able to induce the accumulation of goblet cells in the airways with increased expression of GOB5 and MUC5AC (Fig 8H and 8I). Despite the reduced expression of several Th2-associated asthma genes, IFN-γ treatment induced AHR, inflammation, and goblet cell hyperplasia.

**Figure 9.**
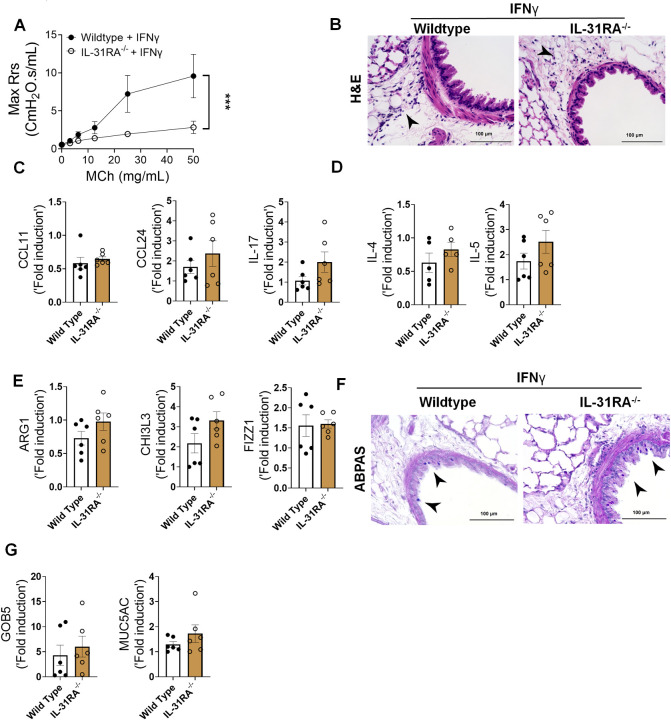
Loss of IL-31RA is sufficient to attenuate IFNγ-induced AHR with no effect on inflammation and goblet cell hyperplasia. **(A)** Wild-type and IL-31RA^−/−^ mice were treated intratracheally with IFN-γ (5 ng) on days D0 and D6 and resistance was measured with increasing doses of methacholine (MCh) using FexiVent. Data are shown as mean ± SEM, n = 6/group. Two-way ANOVA was used, *p<0.05. **(B)** Representative images of hematoxylin and eosin -stained lung sections from wild-type and IL-31RA^−/−^ mice treated with IFN-γ. Images were captured at 20x magnification, scale bar 100 μm, n = 6/group. **(C, D and E)** Quantification of inflammatory cytokines (*CCL11, CCL24* and *IL-17)*, Th2 cytokines (*IL-4* and *IL-5),* and Th2 response-associated genes including *ARG1, CHl3L3,* and *FIZZ1* in the lungs of wild-type and IL-31RA^−/−^ mice treated with IFN-γ. Data are shown as mean ± SEM, n = 6/group. Two-tailed Student’s t-test was used and no statistical signification observed between groups. **(F)** Representative images of alcian blue periodic acid shiff-stained lung sections from wild-type and IL-31RA^−/−^ mice treated with IFN-γ. Images were captured at 20x magnification, scale bar 100μm, n = 6/group. (G) Quantification of mucus-associated genes including *GOB5* and *MUC5AC* transcript levels in the lungs of wild-type and IL-31RA^−/−^ mice treated with IFN-γ. Data are shown as means ± SEM, n = 6/group. Two-tailed Student’s t-test was used, and no statistical signification observed between groups.

To determine whether the expression of IL-31RA was critical for IFN-γ-induced AHR, inflammation, and goblet cell hyperplasia, we intratracheally treated both wild-type and IL31RA^−/−^ mice with IFN-γ. Notably, IFN-γ-induced AHR was significantly attenuated in IL31RA^−/−^ mice compared to that in wild-type mice (Fig 9A and Fig S4). However, as noted with IL-13, increases in peribronchial inflammation and the expression of inflammatory cytokine genes, such as CCL11, CCL24, and IL-17, were similar between wild-type and IL-31RA^−/−^ mice treated with IFN-γ (Fig 9B and 9C). Furthermore, we observed no significant differences in the expression of Th2 cytokine genes (IL-4 and IL-5) or the expression of genes associated with Th2 responses, including ARG1, CHI3L3, and FIZZ1 (Fig 9D and 9E).

To evaluate the effects of IL-31RA deficiency on IFN-γ-induced goblet cell hyperplasia, we performed ABPAS staining of lung sections from wild-type and IL-31RA^−/−^ mice and quantified GOB5 and MUC5AC expression. Analysis of ABPAS-stained lung sections suggested no change in the number of goblet cells that accumulated in the airways of wild-type and IL31RA^−/−^ mice treated with IFNγ (Fig 9F). This finding was further substantiated by quantitative PCR data, which showed no quantitative differences in the expression of GOB5 and MUC5AC with the loss of IL-31RA (Fig 9G). Nevertheless, our *in vivo* studies demonstrated that despite the development of substantial inflammation and mucus hypersecretion, IL-31RA^−/−^ mice showed attenuated AHR in response to either IL-13 or IFN-γ. Overall, our new findings convincingly demonstrate that in allergic asthma, IL-31RA, which is induced by both Th1 and Th2, is critically required for the development of AHR but not inflammation and mucus secretion.

### IL-31RA augments CHRM3-driven calcium signaling in ASMC

**Figure 10.**
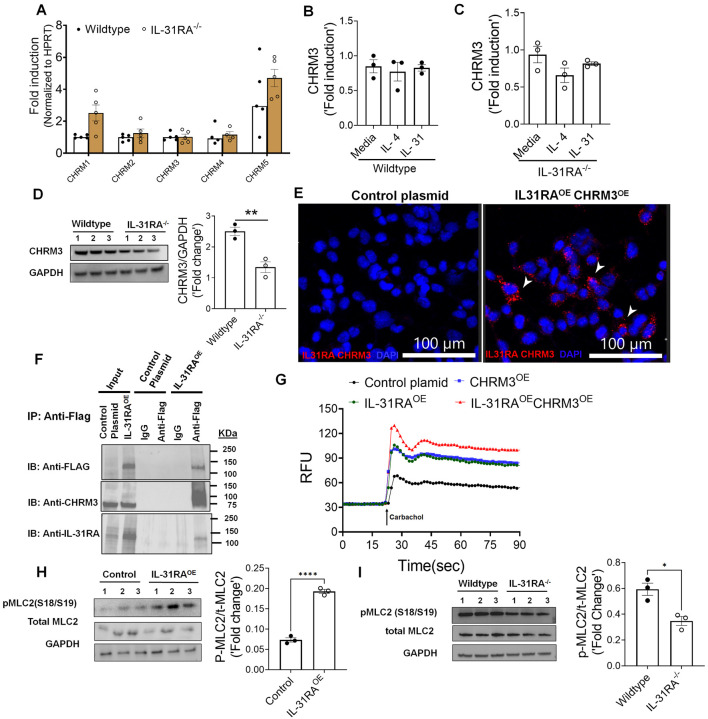
IL-31RA augments CHRM3-driven calcium signaling and MLC phosphorylation in ASMC. **(A)** Quantification of the transcripts of different isoforms of CHRMs in the lungs of wild-type and IL-31RA^−/−^ mice. **(B)** Quantification of the transcripts of CHRM3 in ASMC isolated from wild-type mice and treated with media, IL-4 (10 ng/ml) or IL-31 (500 ng/ml) for 16 h. **(C)** Quantification of the transcripts of CHRM3 in ASMC isolated from IL-31RA^−/−^ mice and treated with media, IL-4 (10 ng/ml) and IL-31 (500 ng/ml) for 16 h. **(D)** ASMC isolated from wild-type and IL-31RA^−/−^ mice were lysed and immunoblotted with antibodies against CHRM3 and GAPDH. CHRM3 protein levels were normalized to GAPDH and shown as fold induced using a bar graph (***P < 0.0005; *n* = 3, Student’s 2-tailed *t* test). (E) HEK293T cells were transiently co-transfected with overexpressing plasmids for CHRM3 and IL-31RA or empty control plasmids for 48 h. The IL-31RA-CHRM3 complex formation was visualized using hybridization probes labeled with Alexa 594 (Red). The nuclei were stained with DAPI (blue) and images were captured at 40X magnification. Scale bar, 100 μm. **(F)** Western blot analyses of CHRM3 in anti-FLAG or control IgG immunoprecipitants from cell lysates of HEK293T cells transiently transfected with a control plasmid or IL-31RA^OE^ plasmid for 72 hrs. Total cell lysates and eluted fractions were immunoblotted with anti-Flag, anti-IL31RA and anti-CHRM3 antibodies. The data presented is a representative of three independent experiments with similar results. **(G)** Increases in carbachol-induced intracellular calcium levels were measured in HEK293T cells transiently transfected with overexpressing plasmids for CHRM3 and IL-31RA or empty control plasmids for 72 h and treated with carbachol (10μM). **(H)** HEK293T cells were transiently co-transfected with overexpressing plasmids for CHRM3 and IL-31RA or empty control plasmids for 48 h and treated with carbachol (10μM) for 10 min. Cell lysates were immunoblotted with antibodies against phospho-MLC, total-MLC and GAPDH. Data are shown as means ± SEM, n = 3/group. **(I)** ASMC isolated from wild-type and IL-31RA^−/−^ mice were treated with carbachol (10μM) for 10 min and cell lysates were immunoblotted with antibodies against phospho-MLC, total-MLC and GAPDH. Data are shown as means ± SEM, n = 3/group

Muscarinic acetylcholine receptors (CHRMs) are predominantly expressed by structural cells such as smooth muscle cells of airways and play a major role in triggering contraction of airways and AHR^55,56^. To evaluate mechanisms by which IL-31RA induces AHR, we measured the transcripts of five major receptor subtypes including CHRM1, CHRM2, CHRM3, CHRM4 and CHRM5 in the lungs of wild-type and IL-31RA^−/−^ mice. Quantification of the lung transcripts suggest no significant differences in the transcript levels of CHRMs in the lungs of IL-31RA^−/−^ mice compared to wild-type mice (Fig 10A). CHRM3 is a dominant receptor subtype expressed in ASMC and coupled to the G_q/11_ family of G proteins to induce calcium signaling and the contraction of ASMC in asthma^55,57^. Therefore, we evaluated the changes in the transcripts of CHRM3 by IL-4 and IL-31 in ASMC of wild-type and IL-31RA^−/−^ mice. Neither IL-4 and IL-31 had a significant effect on the expression of CHRM3 either in the presence or absence of IL-31RA suggesting other mechanisms might be involved in IL-31RA-driven contraction of ASMC (Fig 10B and 10C). To determine post-transcriptional regulation of CHRM3, we measured the protein levels of CHRM3 in AMSC isolated from wild-type and IL-31RA^−/−^ mice. Notably, we observed a significant decrease in the protein levels of CHRM3 in ASMC isolated from IL-31RA^−/−^ mice compared to wild-type mice (Fig 10D). The decrease in CHRM3 protein with no changes in the transcript levels in the absence of IL-31RA may suggest a post-transcriptional stabilization of CHRM3 that may involve a physical interaction between IL-31RA and CHRM3 in ASMC. To identify the complex formation between IL-31RA and CHRM3, we used *in situ* proximity ligation assay (PLA) and measured the complex formation between IL-31RA and CHRM3 in HEK293T cells overexpressing both IL-31RA and CHRM3. We generated overexpression plasmids and our western blot analysis of HEK293T cell lysates show overexpression of IL-31RA and CHRM3 in HEK293T cells (Fig S5). Importantly, we observed bright fluorescent signals corresponding to the IL-31RA-CHRM3 complex formation in HEK293T cells overexpressing IL-31RA and CHRM3 compared to control cells (Fig 10E). By fluorescently labeling plasma membrane with cholera toxin, we demonstrated the colocalization of PLA puncta with the plasma membrane in HEK293T cells overexpressing IL-31RA and CHRM3 compared to control cells (Fig S6). These results demonstrated the specificity of the PLA methodology to detect IL-31RA-CHRM3 complex formation in HEK293T cells overexpressing both IL-31RA and CHRM3 compared to control cells. Next, we assessed whether IL-31RA physically interacts with CHRM3 to augment smooth muscle cell contractility. To assess this, we performed co-immunoprecipitation studies with HEK293T cells transfected with plasmid overexpressing C-terminal FLAG-tagged IL-31RA as these cells naturally express low levels of CHRM3. When we immunoprecipitated FLAG-tagged IL-31RA from cell extracts with antibody against FLAG-tag, endogenous CHRM3 protein was co-purified, as assessed by western blot analysis (Fig 10F). We could not coimmunoprecipitate endogenous CHRM3, a negative control with IgG control or cell lysates from HEK293T cell transfected with control plasmid. Because of the physical interaction between the CHRM3 and IL-31RA, we hypothesized that IL-31RA augments CHRM3-driven calcium signaling. Therefore, we assessed the gain-of-function effects of IL-31RA on CHRM3-driven calcium signaling by carbachol in HEK293T cells. Carbachol treatment in HEK293T cells transfected with control plasmids resulted in elevated intracellular calcium flux (Fig 10G). Notably, overexpression of IL-31RA was sufficient to augment calcium release similar to the levels observed with CHRM3 overexpression. This increase in intracellular calcium release was further elevated in HEK293T cells that co-expressed both IL-31RA and CHRM3 which may suggest a cooperation between IL-31RA and CHRM3 to augment carbachol-induced calcium flux. However, calcium-dependent phosphorylation of myosin light chain (MLC) is a terminal event in the contraction of ASMC^58^. Therefore, we measured carbachol-induced phosphorylation of MLC in HEK293T cells overexpressing IL-31RA compared to control HEK293T cells. Importantly, overexpression of IL-31RA alone was sufficient to augment the levels of carbachol-induced MLC phosphorylation that involved in smooth muscle contraction (Fig 10H). To further demonstrate the positive regulation of MLC phosphorylation by the IL-31RA-CHRM3 axis, we measured carbachol-induced phosphorylation of MLC in ASMC isolated from wild-type and IL-31RA^−/−^ mice. Consistent with reduced CHRM3 expression in IL-31RA deficient ASMC, we observed a significant decrease in carbachol-induced MLC phosphorylation in ASMC isolated from IL-31RA^−/−^ mice compared to wild-type mice (Fig 10I). These results suggest that IL-31RA functions as a positive regulator of CHRM3 and associated carbachol-induced calcium signaling to augment the contractility of ASMC in asthma.

## DISCUSSION

In this study, we investigated the pathophysiological role of the IL-31/IL-31RA axis using two complementary mouse models of allergic asthma. Our Th1/Th2 cytokine intratracheal instillation studies using wild-type and IL-31RA knockout mice revealed an uncoupling of AHR from other airway pathologies typically underlying asthma, including inflammation and goblet cell hyperplasia. Consistent with dominant role for IL-31RA but not IL-31 in asthma pathogenesis in these models, only the receptor for IL-31 was elevated in wild-type mice exposed to allergens, including HDM and SEA, and administration of IL-31 had 1) limited or no effect on MCh-induced AHR, inflammation, and goblet cell hyperplasia and 2) no significant changes in the contraction of airways in the PCLS or collagen gel-embedded ASMCs.

Our findings suggest that the expression of IL-31RA is not essential for both Th1 and Th2 cytokine-induced inflammation and goblet cell hyperplasia. However, this apparently limited role of IL-31RA is consistent with a recently published study in which neutralization of IL-31 had no effect on airway inflammation in wild-type mice that were sensitized and challenged with ovalbumin^59^. Moreover, a study by Neuper et al. demonstrated an elevated expression of IL-31RA but not IL-31 in the lungs of mice challenged with the timothy grass pollen allergen^37^. The role of IL-31RA has been extensively investigated in other disease models, including pulmonary fibrosis and systemic sclerosis. In support of our findings, the loss of IL-31RA had a limited effect on the expression of Th2 cytokines and inflammation but resulted in significant improvement in lung function and pulmonary fibrosis^25,37,60^. Upon repetitive injury with bleomycin, mice deficient in IL-31RA were protected from worsening lung function compared with wild-type mice^37^. However, studies using SEA-induced pulmonary injury model demonstrated elevated granulomatous inflammation and Th2 responses in the absence of IL-31RA^46^. Similarly, studies using the gastrointestinal helminth *Trichuris muris* infection model support elevated Th2 responses and accelerated expulsion of *Trichuris* with significantly decreased worm burdens in the absence of IL-31RA compared with their wild-type counterparts^46,59,61,62^. Therefore, we used an alternative mouse model of allergic asthma, using SEA as an allergen, to assess AHR, airway inflammation, goblet cell hyperplasia, and Th2 responses. Consistent with our findings in the HDM allergen model, we observed no significant differences in SEA-driven Th2 responses between wild-type and IL-31RA deficient mice but observed a significant improvement in AHR. Further, our in vivo studies using intratracheal instillation of IFNγ and IL-13 suggest that IL-31RA-driven AHR could be downstream of both Th1 and Th2 cytokines. The lack of consistency in Th2 responses in the absence of IL-31RA could be due to the disease model used, animal housing conditions, or differences in the generation of IL-31RA knockout mice^37,46,61,62^. Nevertheless, future studies are needed to assess the effect of smooth muscle cell-specific IL-31RA deficiency on AHR and inflammation using both helminth infection and allergic asthma models. In the present study, our findings demonstrated that the loss of IL-31RA is sufficient to attenuate both IFN-γ- and IL-4/IL-13-induced AHR without inhibiting the infiltration of inflammatory cells in the lung. However, mechanistic studies are warranted to determine the molecular pathways through which IL-31RA alters the contractile functions of ASMC and AHR in asthma. These studies should be complemented by the assessment of the potential role of both soluble and membrane associated isoforms of IL-31RA in augmenting the contractile function of ASMC and AHR in asthma^52^.

IFN-γ is a potent Th1 cytokine primarily produced by Th1 T cells that is known to induce AHR in asthma^12,63,64^. Patients with severe asthma who respond poorly to corticosteroid therapy have been shown to have dominant Th1 responses, including elevated IFN-γ in their lungs^12,65^. In vivo studies using animal models of severe asthma have suggested that IFN-γ, but not IL-17, is responsible for heightened AHR^12^. In another study, the blockade of IFN-γ attenuated Th1-cell-induced AHR, and this improvement in AHR appeared to be independent of neutrophils^63^. Similarly, multiple preclinical and clinical studies using antibody-mediated blockade of IL-4/IL-13 signaling have demonstrated the pathogenic role of Th2 cytokines in the development of AHR in allergic asthma^18,50,66^. However, the mechanisms underlying Th1/Th2 cytokine- driven AHR remain unclear. Although the hypothesis tested in this study was straightforward, it was surprising that IL-31RA, but not the Th2 T cell-derived cytokine, IL-31 was responsible for inducing AHR in allergic asthma. Nonetheless, we demonstrated that both Th1 and Th2 cytokines upregulated IL-31RA in ASMC and in the two complementary mouse models of allergic asthma. Our findings on the effect of IFN-γ-induced IL-31RA expression are supported by previous studies in which IFN-γ was shown to induce the expression of IL-31RA in dendritic cells, lung epithelial cells, and fibroblasts^26,31,67,68^. A recent study by Kobayashi *et al*. also demonstrated the role of IFN-γ in the induction of AHR without significantly affecting airway inflammation using intranasal administration of IFN-γ in mice^53^. However, future studies are required to determine mechanisms that uncouple AHR from inflammation and goblet cell hyperplasia in asthma.

We also provide a novel finding that IL-31RA functions as a positive regulator of CHRM3-driven calcium signaling and contractility of ASMC. This is the first study, to our knowledge to show that the IL-31RA-CHRM3 axis induces the phosphorylation of MLC in ASMC. CHRM3 is a key muscarinic receptor expressed by smooth muscle cells in the airways and plays a major role in the contractility of smooth muscle cells in response to muscarinic ligands such as acetylcholine and methacholine^56,58^. CHRM3 is a member of class A GPCR that can mediate ASMC contraction through both calcium-dependent and calcium-independent mechanisms^55,58^. The calcium-dependent smooth muscle cell contractility is dependent on the activation of a subunit of Gq and the release of inositol 1,4,5-triphosphate by phospholipase C^69^. Our studies using both the loss of function and gain-of-function studies suggest that IL-31RA augments CHRM3-driven calcium release and phosphorylation of MLC. Notably, we observed a possible physical interaction between IL-31RA and CHRM3 that may contribute to enhanced calcium signaling and contractility of ASMC through stabilizing CHRM3 protein. It is possible other mechanisms that involve stabilizing CHRM3 or other proteins in macromolecular complexes that involved in calcium-dependent and calcium-independent ASMC contraction^55,58^. How these observations including the physical interaction between IL-31RA and CHRM3 are linked to elevated CHRM3-driven signaling remains unknown. Further studies are required to determine the validity of the hypothesis that IL-31RA stabilizes CHRM3 to augment intracellular calcium signaling in the contraction of ASMC and whether it extends to other CHRMs. Nevertheless, given the robust increases in CHRM3-driven calcium signaling, and ASMC contraction, it is easy to envision the scope of the IL-31RA-CHRM3 axis in inducing AHR in asthma. Our findings provide evidence of the potential therapeutic benefits of targeting IL-31RA, which is downstream of both Th1 and Th2 cytokines, to attenuate AHR. This hypothesis is important for further investigation because therapeutic antibodies targeting IL-31RA are currently in multiple phase II clinical trials for atopic dermatitis^70^. Given these findings, it is important to determine whether neutralizing antibodies or small-molecule inhibitors of IL-31RA can alter muscarinic signaling in ASMC. If so, therapeutic efficacy might be improved with these antibodies and new inhibitors, which may reduce the expression of IL-31RA and/or muscarinic signaling in ASMC. However, even highly effective anti-AHR therapies sufficient to inhibit IL-31RA-driven contraction of ASMC may need to be combined with therapeutics that mitigate inflammation and goblet cell hyperplasia.

In summary, we have described the role of IL-31RA in the induction of AHR, with limited effects on airway inflammation and goblet cell hyperplasia, and this role is independent of its ligand IL-31. Importantly, we identified a novel mechanism underlying ASMC contractility that involves IL-31RA dependent increases in CHRM3-driven calcium signaling and phosphorylation of MLC. Together, these results suggest an important role for IL-31RA in the regulation of AHR and identifying the molecular events underlying IL-31RA-driven AHR may lead to the development of novel therapeutic approaches against AHR in allergic asthma.

## MATERIALS AND METHODS

### Animals.

Wild-type and interleukin receptor IL-31RA^−/−^ mice with a C57BL/6 background were used for in vivo experiments. Age-matched male and female mice of 12–16 weeks of age were used for experiments with at least two repeats. Wild-type mice were obtained from the Jackson Laboratory and maintained in animal facilities. IL-31RA^−/−^ mice used in this study were previously described^26,37,59^. A functional loss of IL-31RA signaling has been induced in these mice through targeted deletion of 4–6 exons in the mouse genome, which encodes the cytokine-binding domain 2 of IL-31RA. All mice were housed under specific pathogen-free conditions at the Cincinnati Children’s Hospital Medical Center, a medical facility approved by American Association for the Accreditation of Laboratory Animal Care. All experimental procedures were approved by Animal Care and Use Committee of Cincinnati Children’s Hospital Medical Center.

### HDM- and SEA-induced allergic asthma mouse models.

Mice were challenged with HDM extracts (*Dermatophagoides pteronyssinus,* Greer laboratories InC), a clinically important and commonly used allergen to induce asthma without the need for an adjuvant. Mice were sensitized twice with 200 μg of HDM in 200 μL of phosphate-buffered saline (PBS) through intraperitoneal injections on days 0 and 7. Mice were further challenged on days 14 and 16 through intratracheal instillation of 50 μg of HDM in 50 μL PBS. Mice were anesthetized with ketamine and xylazine to administer the allergen to the airways. Twenty-four hours following the final challenge with HDM, mice were deeply anesthetized to assess the AHR using Flexivent, and tissues were collected for biochemical and histological analyses. Control mice were sensitized with HDM and challenged with PBS.

The SEA prepared from *Schistosoma mansoni* helminth was used in other experiments to induce AHR and inflammation in mice. SEA has been used for decades as an antigen or allergen owing to its robust induction of type 2 immune responses in animal models. Previous studies have described SEA-induced asthma model^28,38^. Briefly, mice were sensitized with 10 μg of SEA in 200 μL by intraperitoneal injection on days 0 and 14. Two weeks later, mice were anesthetized with ketamine and xylazine, and then challenged by intratracheal instillation with 10 μg of SEA in 50 μl of PBS, twice on days 28 and 31. The day following the last challenge, the mice were euthanized and samples were collected for further analysis.

### Cytokine treatments.

To evaluate the specific role of cytokines in the induction of AHR and inflammation, wild-type and IL-31RA^−/−^ mice were intratracheally administered recombinant mouse cytokines, as previously described with some modifications^18,20^. Mice were anesthetized by an intraperitoneal injection of ketamine/xylazine, rIL-31 (10 μg, R&D Systems), rIFN-γ (5 μg, R&D Systems), or rIL-13 (5 μg, R&D Systems) and intratracheally instilled in 50 μL of saline on days 0 and 6. Twenty-four hours after the last challenge, mice were euthanized, airway resistance was measured using Flexivent, and the lungs were collected to assess histological changes and gene expression analysis using reverse transcriptase PCR (RT-PCR). Control mice were treated with a saline solution.

### Preparation of BAL cells.

Twenty-four hours after the last challenge with the allergen, the mice were anesthetized, and the lungs were lavaged with 1 mL of ice-cold and sterile PBS using a 1 mL insulin syringe through a tracheal catheter. BAL fluid was centrifuged for 5 min at 250 × *g* at 4 °C. Cell pellets containing BAL cells were resuspended in 500 μL of PBS. Total cell counts were determined using an automated cell counter (Thermo Fisher Scientific, Waltham, MA, USA). BAL cells were used for cytospin preparation and stained with the Diff-Quick staining kit (Thermo Fisher). Differential cell counts were determined using morphological criteria under a microscope.

### Histology.

Mice were euthanized 24 h after the last allergen (HDM or SEA) challenge or after the last cytokine instillation. The lungs were inflated, fixed in 10% neutral formalin, and collected for histological analysis. Paraffin-embedded 5 μm sections of the left lobe were stained with H&E to examine associated airway inflammation and peribronchial cell infiltration. ABPAS (Alcian blue periodic acid Schiff) staining was used to assess goblet cell hyperplasia and mucus hypersecretion. Goblet cells were further counted on lung sections and expressed as the percentage of ABPAS-positive cells of the total nucleated cells of the airway epithelium, using Metamorph imaging software (Molecular Devices). At least 10 images per mouse were used to count goblet and total epithelial cells^68^. Images were captured at 40x magnification using a Keyence BZ-X microscope with a focus on the airway areas to quantify the ABPAS-positive cells.

### AHR measurements.

AHR was assessed in vivo using the forced oscillation technique in FlexiVent system (SCIREQ, Montreal, QC, Canada). Twenty-four hours after the last challenge, the mice were anesthetized using a ketamine/xylazine mix. Tracheotomy was performed, and the trachea was cannulated using a G20 stainless catheter and connected to the FlexiVent machine for airway resistance measurements. Increasing doses of methacholine (MCh, Sigma Aldrich), 0, 3, 6, 12.5, 25, and 50 mg/mL in PBS, were aerosolized using an ultrasonic nebulizer connected to the FlexiVent system and administrated following the manufacturer’s instructions. Lung function measurements were recorded, and airway resistances were presented as the maximum resistance (Max Rrs) values recorded for increasing MCh doses.

### PCLS model.

PCLS were prepared as described previously^37,71^. Eight mice of 10 weeks of age were euthanized with a lethal dose of sodium pentobarbital. The lungs were inflated with warm low melting agarose at 37 °C through a tracheal cannula, followed by 0.2 mL of air to flush the agarose. The left lung and apical and basal right lobes were used to generate PCLS. Lungs were sliced into 300 μm section in 4 °C cold HBSS without calcium using a VF-310Z vibratome (Pecisionary Instruments, Winchester, MA, USA). PCLS were collected in DMEM media without serum, and the media were changed at least four times with intermittent shaking to remove residual agarose and incubated in a humidified incubator with 5% CO_2_ at 37 °C. On the following day, the media was replaced with fresh media without serum for the same-day experiment. For cytokine treatments, PCLS were treated with IL-13 (50 ng/mL, R&D Systems) or IL-31 (500 ng/mL, R&D Systems) for 24 h in a low-serum media (1% FBS) prior to the contraction assay using increasing doses of MCh. Lung slices were allowed to rest and relax before the next dose of MCh was administered to the samples. Changes in the airway lumen area were recorded in a time-lapse setting with images captured every 5 s for 5 min following each dose of MCh using a 10x objective on a temperature-controlled Nikon inverted microscope at 37 °C with 5% CO_2_ (NIKON Ti2 widefield, Japan). Airway areas were analyzed using NIKON NIS-Elements analysis software, and changes were expressed as the percentage area of contraction compared to the initial baseline area.

### RNA isolation and real-time PCR.

Lung tissues were dissociated using TRIzol (Life Technologies) using beads and a high-speed homogenizer (Thermo Fisher). RNA was isolated from lung tissues, primary mouse ASMs, or primary asthmatic human bronchial ASMC (Lonza, Walkerville,MD, USA) using an RNAeasy mini kit (QIAGEN) following the manufacturer’s instructions and as previously described in our previous studies^28,72^. cDNA was synthesized using Superscript III (ThermoFisher), and real-time PCR was performed using SYBR Select Master Mix (Bio-Rad) and a CFX384 Touch Real-Time PCR instrument (Bio-Rad). Data were analyzed using CFX Maestro software version 4.0. Target gene transcripts were normalized using hypoxanthine-guanine phosphor ribosyl transferase (HPRT, for murine cells and tissues) or β-actin (for human cells) as housekeeping genes. The list of primers used to measure the transcript levels of the genes of interest can be found in the supplementary material (Table S1).

### Collagen gel contraction assay.

Human bronchial smooth muscle cells were obtained from Lonza (Wakersville, MD, USA), and murine ASMC were prepared via enzymatic digestion of the mouse trachea at 37 °C for 60 min, as previously described^73^. A single-cell suspension of digested trachea was seeded on a 100 mm petri dish (n = 4/dish) using Hams/F12 culture media supplemented with 10% FBS and antibiotics. Spindle-shaped cells were allowed to grow to 80% confluence, and cells from passages 1–2 were used for the collagen gel contraction assay. ASMCs were seeded into rat tail collagen gel matrices as described previously^74,75^. To determine the effect of cytokines on ASMC contraction, cells were treated with cytokines (rIL-31 500 ng/mL or rIL-13, 50 ng/mL) or media for 48 h prior to embedding them in collagen gel. Collagen gels were prepared using cells treated with cytokines and media and grown under similar conditions in Ham’s/F12 complete media. The collagen gels were detached from the walls, and images were captured at 0, 12 or 16, 24, and 48 h using a stereo microscope. The area of contraction was measured using ImageJ software and described as the percentage of the contracted area compared with the baseline area of the gel.

### Western Blotting.

Western blot analysis of smooth muscle cell lysates were performed as described earlier^72^. Briefly, cell lysates were prepared using the RIPA lysis buffer containing protease and phosphatase inhibitors. After SDS–PAGE separation, proteins were transferred to nitrocellulose membrane and then were probed using specific primary antibodies CHRM3(1:1000; ab126168), phosphoMLC (1:1000; CST 3771S), totalMLC (1:1000; CST3672s), and GAPDH (1:2000 Bethyl A300–643A) followed by detection with HRP conjugated secondary antibodies. Bands were quantified using the volume integration function of the imager software, (BIORAD).

### Calcium flux assay.

HEK293T cells were transiently transfected with overexpressing plasmids for IL-31RA, CHRM3 or empty control plasmids and cultured in DMEM media for 72 h (Fig S4). On day 3, cells were loaded with Fluo-4 AM (5μM) (Invitrogen) for one hour and stimulated with carbachol (10 μm) to measure changes in the fluorescence in real time for 90 sec using a Flex Station III (Molecular Devices).

### *In situ* proximity ligation assay.

HEK293T cells were transiently transfected with overexpressing plasmids for IL-31RA (Origene, RC218212L1) and CHRM3 (Origene, SC119736) or control empty plasmids (pLJM1-EGFP, Addgene 19319 and pLenti-C-Myc-DDK-P2A-Puro, Origene PS100092) using Lipofectamine 3000 and cultured for 48 h. Then, cells were fixed for 5 min in ice cold methanol at 4°C and incubated with Duolink blocking solution (Sigma) for 1 hour at room temperature followed by overnight incubation with primary antibodies for IL-31RA (15 μg/ml, R&D Systems AF2769) and CHRM3(1:500, Abcam ab126168). The incubation was followed by three 5 min washes and incubation with PLA probes anti-goat PLUS (DUO92003) and anti-rabbit MINUS (DUO92005) for 1 h at 37°C as recommended in the Duolink PLA kit (Sigma). After three washes and cells were incubated with the hybridization solution containing DNA ligase for 30 min at 37°C. Cells were washed and incubated with the amplification-polymerase at 37°C for 100 min, washed three times and mounted with DAPI mounting medium. To identify the membrane localization of *in situ* PLA signals, the cells were counterstained with cholera toxin conjugated with Alexa Fluor 488 (1:200, Thermo Fischer). Fluorescence images were acquired using a Nikon A1R confocal laser scanning microscope and analyzed using Imaris image analysis software.

### Immunoprecipitation Studies.

HEK293T cells were transiently transfected with overexpressing plasmids for IL-31RA (Origene) or empty control plasmid using Lipofectamine 3000 and cultured for 72 h. Cell lysates were prepared in RIPA buffer supplemented with Protease inhibitor cocktail and incubated with agarose A/G beads conjugated with anti-FLAG or control IgG antibodies. Immunoprecipitants were collected by centrifugation, washed three times with PBS, eluted with low pH glycine buffer and subjected to SDS-PAGE with Laemmli sample buffer. Membranes were immunoblotted with anti-Flag, anti-IL31RA and anti-CHRM3 antibodies followed by detection with HRP conjugated secondary antibodies and visualized with ECL on a ChemiDOC Touch imaging system (Bio-Rad).

### Statistical Analysis.

Data were analyzed using Prism (version 9; GraphPad, San Diego, CA, USA). Quantitative data are presented as bars with individual dots, including mean ± SEM. Data were considered statistically significant when the p-values were less than 0.05. Two tailed Student’s *t*-test was used to compare the means between the two groups. For multiple comparisons, one-way ANOVA with post hoc Tukey’s test or 2-way ANOVA with post hoc Sidak’s test was used.

## Data Availability

All data are available in the main text or the Supplementary Materials.

## References

[1] Belova A., Fann N., Haskell J., Hubbell B. & Narayan T. Estimating Lifetime Cost of Illness. An Application to Asthma. Ann Am Thorac Soc 17, 1558–1569 (2020). 10.1513/AnnalsATS.201910-729OC 32931705 PMC13288218

[2] Czira A. A systematic literature review of burden of illness in adults with uncontrolled moderate/severe asthma. Respir Med 191, 106670 (2022). 10.1016/j.rmed.2021.106670 34883444

[3] Reddel H. K. An official American Thoracic Society/European Respiratory Society statement: asthma control and exacerbations: standardizing endpoints for clinical asthma trials and clinical practice. Am J Respir Crit Care Med 180, 59–99 (2009). 10.1164/rccm.200801-060ST 19535666

[4] Papi A., Brightling C., Pedersen S. E. & Reddel H. K. Asthma. Lancet 391, 783–800 (2018). 10.1016/S0140-6736(17)33311-1 29273246

[5] Scichilone N., Battaglia S., La Sala A. & Bellia V. Clinical implications of airway hyperresponsiveness in COPD. Int J Chron Obstruct Pulmon Dis 1, 49–60 (2006). 10.2147/copd.2006.1.1.49 18046902 PMC2706603

[6] Levine H. Reversible airway obstruction in cystic fibrosis: Common, but not associated with characteristics of asthma. J Cyst Fibros 15, 652–659 (2016). 10.1016/j.jcf.2016.01.003 26826913

[7] Kuruvilla M. E., Lee F. E. & Lee G. B. Understanding Asthma Phenotypes, Endotypes, and Mechanisms of Disease. Clin Rev Allergy Immunol 56, 219–233 (2019). 10.1007/s12016-018-8712-1 30206782 PMC6411459

[8] Ashino S. A T(h)17-polarized cell population that has infiltrated the lung requires cells that convert to IFN-{gamma} production in order to induce airway hyperresponsiveness. Int Immunol 22, 503–513 (2010). 10.1093/intimm/dxq034 20501609

[9] Chambers E. S. Distinct endotypes of steroid-resistant asthma characterized by IL-17A(high) and IFN-gamma(high) immunophenotypes: Potential benefits of calcitriol. J Allergy Clin Immunol 136, 628–637 e624 (2015). 10.1016/j.jaci.2015.01.026 25772594 PMC4559139

[10] Kudo M. IL-17A produced by alphabeta T cells drives airway hyper-responsiveness in mice and enhances mouse and human airway smooth muscle contraction. Nat Med 18, 547–554 (2012). 10.1038/nm.2684 22388091 PMC3321096

[11] Munitz A., Brandt E. B., Mingler M., Finkelman F. D. & Rothenberg M. E. Distinct roles for IL-13 and IL-4 via IL-13 receptor alpha1 and the type II IL-4 receptor in asthma pathogenesis. Proc Natl Acad Sci U S A 105, 7240–7245 (2008). 10.1073/pnas.0802465105 18480254 PMC2386078

[12] Raundhal M. High IFN-gamma and low SLPI mark severe asthma in mice and humans. J Clin Invest 125, 3037–3050 (2015). 10.1172/JCI80911 26121748 PMC4563754

[13] Sugimoto T. Interleukin 18 acts on memory T helper cells type 1 to induce airway inflammation and hyperresponsiveness in a naive host mouse. J Exp Med 199, 535–545 (2004). 10.1084/jem.20031368 14970180 PMC2211833

[14] Becerra-Diaz M., Wills-Karp M. & Heller N. M. New perspectives on the regulation of type II inflammation in asthma. F1000Res 6, 1014 (2017). 10.12688/f1000research.11198.1 28721208 PMC5497827

[15] Gour N. & Wills-Karp M. IL-4 and IL-13 signaling in allergic airway disease. Cytokine 75, 68–78 (2015). 10.1016/j.cyto.2015.05.014 26070934 PMC4532591

[16] Wynn T. A. Type 2 cytokines: mechanisms and therapeutic strategies. Nat Rev Immunol 15, 271–282 (2015). 10.1038/nri3831 25882242

[17] Tliba O. IL-13 enhances agonist-evoked calcium signals and contractile responses in airway smooth muscle. Br J Pharmacol 140, 1159–1162 (2003). 10.1038/sj.bjp.0705558 14597600 PMC1574143

[18] Wills-Karp M. Interleukin-13: central mediator of allergic asthma. Science 282, 2258–2261 (1998). 10.1126/science.282.5397.2258 9856949

[19] Finkelman F. D. Suppressive effect of IL-4 on IL-13-induced genes in mouse lung. J Immunol 174, 4630–4638 (2005). 10.4049/jimmunol.174.8.4630 15814686

[20] Perkins C., Wills-Karp M. & Finkelman F. D. IL-4 induces IL-13-independent allergic airway inflammation. J Allergy Clin Immunol 118, 410–419 (2006). 10.1016/j.jaci.2006.06.004 16890766

[21] Guttman-Yassky E. Dupilumab progressively improves systemic and cutaneous abnormalities in patients with atopic dermatitis. J Allergy Clin Immunol 143, 155–172 (2019). 10.1016/j.jaci.2018.08.022 30194992

[22] Rabe K. F. Efficacy and Safety of Dupilumab in Glucocorticoid-Dependent Severe Asthma. N Engl J Med 378, 2475–2485 (2018). 10.1056/NEJMoa1804093 29782224

[23] Menzella F. Severe refractory asthma: current treatment options and ongoing research. Drugs Context 7, 212561 (2018). 10.7573/dic.212561 30534175 PMC6284776

[24] Singh B. IL-31-Driven Skin Remodeling Involves Epidermal Cell Proliferation and Thickening That Lead to Impaired Skin-Barrier Function. PLoS One 11, e0161877 (2016). 10.1371/journal.pone.0161877 27556734 PMC4996532

[25] Yaseen B. Interleukin-31 promotes pathogenic mechanisms underlying skin and lung fibrosis in scleroderma. Rheumatology (Oxford) 59, 2625–2636 (2020). 10.1093/rheumatology/keaa195 32365362

[26] Dillon S. R. Interleukin 31, a cytokine produced by activated T cells, induces dermatitis in mice. Nat Immunol 5, 752–760 (2004). 10.1038/ni1084 15184896

[27] Nobbe S. IL-31 expression by inflammatory cells is preferentially elevated in atopic dermatitis. Acta Derm Venereol 92, 24–28 (2012). 10.2340/00015555-1191 22041865

[28] Yombo D. J. K., Mentink-Kane M. M., Wilson M. S., Wynn T. A. & Madala S. K. Heat shock protein 70 is a positive regulator of airway inflammation and goblet cell hyperplasia in a mouse model of allergic airway inflammation. J Biol Chem 294, 15082–15094 (2019). 10.1074/jbc.RA119.009145 31431507 PMC6791332

[29] Chattopadhyay S. Interleukin-31 and oncostatin-M mediate distinct signaling reactions and response patterns in lung epithelial cells. J Biol Chem 282, 3014–3026 (2007). 10.1074/jbc.M609655200 17148439

[30] Zhang Q., Putheti P., Zhou Q., Liu Q. & Gao W. Structures and biological functions of IL-31 and IL-31 receptors. Cytokine Growth Factor Rev 19, 347–356 (2008). 10.1016/j.cytogfr.2008.08.003 18926762 PMC2659402

[31] Jawa R. S. Regulated expression of the IL-31 receptor in bronchial and alveolar epithelial cells, pulmonary fibroblasts, and pulmonary macrophages. J Interferon Cytokine Res 28, 207–219 (2008). 10.1089/jir.2007.0057 18439099

[32] Nemmer J. M. Interleukin-31 Signaling Bridges the Gap Between Immune Cells, the Nervous System and Epithelial Tissues. Front Med (Lausanne) 8, 639097 (2021). 10.3389/fmed.2021.639097 33644104 PMC7902767

[33] Raap U. Human basophils are a source of - and are differentially activated by - IL-31. Clin Exp Allergy 47, 499–508 (2017). 10.1111/cea.12875 28000952

[34] Yagi Y. Interleukin-31 stimulates production of inflammatory mediators from human colonic subepithelial myofibroblasts. Int J Mol Med 19, 941–946 (2007). 17487427

[35] Lai T. Interleukin-31 expression and relation to disease severity in human asthma. Sci Rep 6, 22835 (2016). 10.1038/srep22835 26956917 PMC4783779

[36] Lei Z. SCF and IL-31 rather than IL-17 and BAFF are potential indicators in patients with allergic asthma. Allergy 63, 327–332 (2008). 10.1111/j.1398-9995.2007.01566.x 18053009

[37] Yombo D. J. K., Odayar V., Gupta N., Jegga A. G. & Madala S. K. The Protective Effects of IL-31RA Deficiency During Bleomycin-Induced Pulmonary Fibrosis. Front Immunol 12, 645717 (2021). 10.3389/fimmu.2021.645717 33815402 PMC8017338

[38] Edukulla R. Th2 Cytokines Augment IL-31/IL-31RA Interactions via STAT6-dependent IL-31RA Expression. J Biol Chem 290, 13510–13520 (2015). 10.1074/jbc.M114.622126 25847241 PMC4505597

[39] Perkins C. Selective stimulation of IL-4 receptor on smooth muscle induces airway hyperresponsiveness in mice. J Exp Med 208, 853–867 (2011). 10.1084/jem.20100023 21464224 PMC3135339

[40] McKnight C. G., Potter C. & Finkelman F. D. IL-4Ralpha expression by airway epithelium and smooth muscle accounts for nearly all airway hyperresponsiveness in murine allergic airway disease. Mucosal Immunol 13, 283–292 (2020). 10.1038/s41385-019-0232-7 31745261 PMC7641252

[41] Cates E. C. Intranasal exposure of mice to house dust mite elicits allergic airway inflammation via a GM-CSF-mediated mechanism. J Immunol 173, 6384–6392 (2004). 10.4049/jimmunol.173.10.6384 15528378

[42] Woo L. N. A 4-Week Model of House Dust Mite (HDM) Induced Allergic Airways Inflammation with Airway Remodeling. Sci Rep 8, 6925 (2018). 10.1038/s41598-018-24574-x 29720689 PMC5932037

[43] Izuhara K. The mechanism of mucus production in bronchial asthma. Curr Med Chem 16, 2867–2875 (2009). 10.2174/092986709788803196 19689269

[44] Lambrecht B. N. & Hammad H. The airway epithelium in asthma. Nat Med 18, 684–692 (2012). 10.1038/nm.2737 22561832

[45] Ordonez C. L. Mild and moderate asthma is associated with airway goblet cell hyperplasia and abnormalities in mucin gene expression. Am J Respir Crit Care Med 163, 517–523 (2001). 10.1164/ajrccm.163.2.2004039 11179133

[46] Perrigoue J. G. IL-31-IL-31R interactions negatively regulate type 2 inflammation in the lung. J Exp Med 204, 481–487 (2007). 10.1084/jem.20061791 17353366 PMC2137900

[47] Fritz D. K. A mouse model of airway disease: oncostatin M-induced pulmonary eosinophilia, goblet cell hyperplasia, and airway hyperresponsiveness are STAT6 dependent, and interstitial pulmonary fibrosis is STAT6 independent. J Immunol 186, 1107–1118 (2011). 10.4049/jimmunol.0903476 21160052

[48] Lin Y. L., Chen S. H. & Wang J. Y. Critical role of IL-6 in dendritic cell-induced allergic inflammation of asthma. J Mol Med (Berl) 94, 51–59 (2016). 10.1007/s00109-015-1325-8 26232935

[49] Rincon M. & Irvin C. G. Role of IL-6 in asthma and other inflammatory pulmonary diseases. Int J Biol Sci 8, 1281–1290 (2012). 10.7150/ijbs.4874 23136556 PMC3491451

[50] Ramalingam T. R. Unique functions of the type II interleukin 4 receptor identified in mice lacking the interleukin 13 receptor alpha1 chain. Nature immunology 9, 25–33 (2008). https://doi.org/10.1038/ni1544 [pii] 10.1038/ni1544 18066066 10.1038/ni1544 PMC2692551

[51] Wilson M. S. Suppression of murine allergic airway disease by IL-2:anti-IL-2 monoclonal antibody-induced regulatory T cells. J Immunol 181, 6942–6954 (2008). 10.4049/jimmunol.181.10.6942 18981114 PMC2706157

[52] Maier E. Prerequisites for Functional Interleukin 31 Signaling and Its Feedback Regulation by Suppressor of Cytokine Signaling 3 (SOCS3). J Biol Chem 290, 24747–24759 (2015). 10.1074/jbc.M115.661306 26306032 PMC4598987

[53] Kobayashi M. IFN-gamma elevates airway hyper-responsiveness via upregulation of neurokinin A/neurokinin-2 receptor signaling in a severe asthma model. Eur J Immunol 42, 393–402 (2012). 10.1002/eji.201141845 22105467

[54] Xu J. The Cytokine TGF-beta Induces Interleukin-31 Expression from Dermal Dendritic Cells to Activate Sensory Neurons and Stimulate Wound Itching. Immunity 53, 371–383 e375 (2020). 10.1016/j.immuni.2020.06.023 32673566 PMC7362873

[55] Fryer A. D. & Jacoby D. B. Muscarinic receptors and control of airway smooth muscle. Am J Respir Crit Care Med 158, S154–160 (1998). 10.1164/ajrccm.158.supplement_2.13tac120 9817739

[56] Bradley S. J. Mapping physiological G protein-coupled receptor signaling pathways reveals a role for receptor phosphorylation in airway contraction. Proc Natl Acad Sci U S A 113, 4524–4529 (2016). 10.1073/pnas.1521706113 27071102 PMC4843461

[57] Dowal L., Provitera P. & Scarlata S. Stable association between G alpha(q) and phospholipase C beta 1 in living cells. J Biol Chem 281, 23999–24014 (2006). 10.1074/jbc.M512330200 16754659

[58] Gosens R., Zaagsma J., Meurs H. & Halayko A. J. Muscarinic receptor signaling in the pathophysiology of asthma and COPD. Respir Res 7, 73 (2006). 10.1186/1465-9921-7-73 16684353 PMC1479816

[59] Bilsborough J., Mudri S., Chadwick E., Harder B. & Dillon S. R. IL-31 receptor (IL-31RA) knockout mice exhibit elevated responsiveness to oncostatin M. J Immunol 185, 6023–6030 (2010). 10.4049/jimmunol.0902769 20956341

[60] Kuzumi A. Interleukin-31 promotes fibrosis and T helper 2 polarization in systemic sclerosis. Nat Commun 12, 5947 (2021). 10.1038/s41467-021-26099-w 34642338 PMC8511151

[61] Huang J. IL-31 plays dual roles in lung inflammation in an OVA-induced murine asthma model. Biol Open 8 (2019). 10.1242/bio.036244 PMC6361213 30647024

[62] Neuper T. IL-31 transgenic mice show reduced allergen-induced lung inflammation. Eur J Immunol 51, 191–196 (2021). 10.1002/eji.202048547 32648940 PMC7818168

[63] Cui J. TH1-mediated airway hyperresponsiveness independent of neutrophilic inflammation. J Allergy Clin Immunol 115, 309–315 (2005). 10.1016/j.jaci.2004.10.046 15696086

[64] Hayashi N. T helper 1 cells stimulated with ovalbumin and IL-18 induce airway hyperresponsiveness and lung fibrosis by IFN-gamma and IL-13 production. Proc Natl Acad Sci U S A 104, 14765–14770 (2007). 10.1073/pnas.0706378104 17766435 PMC1976243

[65] Kim Y. S. IL-12-STAT4-IFN-gamma axis is a key downstream pathway in the development of IL-13-mediated asthma phenotypes in a Th2 type asthma model. Exp Mol Med 42, 533–546 (2010). 10.3858/emm.2010.42.8.054 20592486 PMC2928926

[66] Grunig G. Requirement for IL-13 independently of IL-4 in experimental asthma. Science 282, 2261–2263 (1998). 10.1126/science.282.5397.2261 9856950 PMC3897229

[67] Feld M., Shpacovitch V. M., Fastrich M., Cevikbas F. & Steinhoff M. Interferon-gamma induces upregulation and activation of the interleukin-31 receptor in human dermal microvascular endothelial cells. Exp Dermatol 19, 921–923 (2010). 10.1111/j.1600-0625.2010.01147.x 20849534

[68] Lou Y. P. Platelet-activating factor induces goblet cell hyperplasia and mucin gene expression in airways. Am J Respir Crit Care Med 157, 1927–1934 (1998). 10.1164/ajrccm.157.6.9709113 9620929

[69] Singer A. U., Waldo G. L., Harden T. K. & Sondek J. A unique fold of phospholipase C-beta mediates dimerization and interaction with G alpha q. Nat Struct Biol 9, 32–36 (2002). 10.1038/nsb731 11753430

[70] Bieber T. Atopic dermatitis: an expanding therapeutic pipeline for a complex disease. Nat Rev Drug Discov 21, 21–40 (2022). 10.1038/s41573-021-00266-6 34417579 PMC8377708

[71] Cooper P. R. 20-HETE mediates ozone-induced, neutrophil-independent airway hyper-responsiveness in mice. PLoS One 5, e10235 (2010). 10.1371/journal.pone.0010235 20422032 PMC2857875

[72] Gajjala P. R. Dysregulated overexpression of Sox9 induces fibroblast activation in pulmonary fibrosis. JCI Insight 6 (2021). 10.1172/jci.insight.152503 PMC8564901 34520400

[73] Balenga N. A., Jester W., Jiang M., Panettieri R. A. Jr. & Druey K. M. Loss of regulator of G protein signaling 5 promotes airway hyperresponsiveness in the absence of allergic inflammation. J Allergy Clin Immunol 134, 451–459 (2014). 10.1016/j.jaci.2014.01.019 24666695 PMC4119844

[74] Ngo P., Ramalingam P., Phillips J. A. & Furuta G. T. Collagen gel contraction assay. Methods Mol Biol 341, 103–109 (2006). 10.1385/1-59745-113-4:103 16799192

[75] Matsumoto H. Comparison of gel contraction mediated by airway smooth muscle cells from patients with and without asthma. Thorax 62, 848–854 (2007). 10.1136/thx.2006.070474 17412779 PMC2094259

